# Recurrent spontaneous *Escherichia coli* meningitis in an adult: a case report

**DOI:** 10.1093/jacamr/dlad029

**Published:** 2023-03-28

**Authors:** Anne V Amulele, Gerald Ong’ayo, Alfred M Arara, Edwin W Machanja, Anthony Etyang, Nadia A Aliyan, David W Wareham, James A Berkley, Nicola C Gordon

**Affiliations:** Epidemiology and Demography Department, KEMRI Wellcome Trust Research Programme, Kilifi, Kenya; Epidemiology and Demography Department, KEMRI Wellcome Trust Research Programme, Kilifi, Kenya; Epidemiology and Demography Department, KEMRI Wellcome Trust Research Programme, Kilifi, Kenya; Clinical Research Department, KEMRI Wellcome Trust Research Programme, Kilifi, Kenya; Epidemiology and Demography Department, KEMRI Wellcome Trust Research Programme, Kilifi, Kenya; Kilifi County Hospital, Department of Health, Kilifi, Kenya; Antimicrobial Research Group, Blizard Institute, Queen Mary University of London, London, UK; Clinical Research Department, KEMRI Wellcome Trust Research Programme, Kilifi, Kenya; Nuffield Department of Medicine, Oxford University, Oxford, UK; Rare and Imported Pathogens Laboratory, UK Health Security Agency, Salisbury, UK

## Abstract

**Objectives:**

The aim of this study was to characterize an unusual case of spontaneous, community-acquired *Escherichia coli* meningitis in an adult presenting to a general hospital in Kenya, where initial clinical recovery was followed by reinfection with an MDR, hospital-acquired strain.

**Patient and methods:**

An adult presented to a hospital in Kenya with meningitis symptoms. *E. coli* was cultured from CSF. Treatment with ceftriaxone was successful; however, the patient relapsed a few days later. *E. coli* was cultured from CSF and blood during the reinfection episode, though the patient died during admission. We sequenced the isolates using Illumina MiSeq and performed antimicrobial susceptibility testing, fitness and virulence assays on the bacteria.

**Results:**

The *E. coli* isolates from the two episodes were found to be distinct: the initial strain was ST88, serotype O8 H17 while the subsequent episode was caused by an ST167, serotype O101 H5 MDR strain. The ST88 strain was susceptible to all drugs except ampicillin and amoxicillin/clavulanate while the ST167 strain was MDR, including to all β-lactam drugs due to the presence of the carbapenemase gene *bla*_NDM-5._ The hospital-acquired ST167 strain was also resistant to newer drugs such as cefiderocol and eravacycline, which are currently not available locally, and had overall lower fitness and virulence *in vitro* compared with the initial infecting strain.

**Conclusions:**

Though less fit and virulent *in vitro*, the MDR strain was fatal, suggesting that host factors, rather than bacterial virulence, may have been of greater importance in this patient’s outcome.

## Introduction

The spread of MDR infections is a worldwide health concern and its impact on mortality is particularly high in resource-limited settings^1^ where several factors, including lack of access to reliable laboratory infrastructure for microbiological analysis, delay access to optimally targeted medication.

*Escherichia coli* causes a variety of diseases and is a common cause of urinary tract infections, meningitis, sepsis and abdominal infections, and resistance in the pathogen is a leading cause of resistance-associated mortality.^1^ In adults, spontaneous community-acquired *E. coli* meningitis is rare and carries a high mortality. It is most often associated with neurosurgical procedures or head trauma,^2,3^ with other cases reported as secondary dissemination from a urinary tract or gastrointestinal tract infection.^4,5^ Several risk factors have been identified, including advanced age, chronic alcoholism, liver cirrhosis, immunocompromised conditions due to HIV infection or immunosuppressive therapy and diabetes mellitus or, in some cases, strongyloidiasis infection.^4,6–8^

In this paper, we describe a case of spontaneous onset of meningeal infection by a sensitive *E. coli* strain followed by reinfection with an MDR strain. Both isolates were characterized using WGS and *in vitro* fitness assays to evaluate the contribution of bacterial versus host factors.

## Patients and methods

## Ethical approval

Isolates and clinical details for this case report were collected within a longitudinal surveillance study approved by the Kenya Medical Research Institute (KEMRI) Scientific and Ethics Review Unit (SERU) (SSC 1433). Separate approval was acquired for further data analysis described (SERU 3748). Written informed consent to participate in the surveillance study was obtained from the case on admission to hospital. Publication consent for the case report was obtained from the next of kin.

## Case report

A middle-aged male patient presented to a county general hospital in rural coastal Kenya with a 1 week history of fever, diarrhoea, vomiting, headache and confusion. He reported progressive weight loss over the preceding months with loss of appetite and poor nutrition. He had a history of excessive alcohol use and cigarette smoking but no other significant past medical history. He denied attending any other healthcare facilities in the preceding 6 months and tested negative for HIV. At admission, blood cultures were taken, and a lumbar puncture was performed: the CSF was noted to be turbid, with an elevated protein concentration (3.55 g/L), and WBC count (>999 cells/µL), and a glucose level below the detection limit of the assay. Direct staining of the CSF showed Gram-negative rods, which were confirmed to be *E. coli*, susceptible by disc diffusion to gentamicin, amikacin, chloramphenicol, trimethoprim/sulfamethoxazole, ceftriaxone, ciprofloxacin, ceftazidime, cefoxitin, cefotaxime and imipenem, and with reduced susceptibility to ampicillin and amoxicillin/clavulanate. The blood culture was negative after 72 h. The patient was admitted to a general ward and was treated with 2 g IV ceftriaxone twice daily for 3 weeks. He also received diazepam and IV thiamine to prevent delirium tremens and other overt signs of alcohol withdrawal. He made a full clinical recovery with resolution of fever and meningism and was discharged from the general service. However, he delayed leaving the hospital and was retained in the general ward due to financial reasons. Three days after completing the ceftriaxone course, he once more became unwell with fever and reduced level of consciousness. He had a repeat lumbar puncture and blood culture, and ceftriaxone was restarted empirically. This time, the CSF was clear on examination, with a protein concentration of 2.67 g/L, WBC count of 760 cells/µL and glucose again below the detection limit of the assay. Within 72 h, both the blood culture and CSF grew a carbapenem-resistant *E. coli* susceptible to gentamicin, amikacin, co-trimoxazole and chloramphenicol. He was switched to chloramphenicol but unfortunately died 2 days later.

Three *E. coli* strains, from CSF culture at admission (ECO-1) and during recurrence (ECO-2) and from blood during recurrence (ECO-3), were obtained from the patient and studies were undertaken to characterize virulence and resistance factors.

## Laboratory methods

### Antimicrobial susceptibility testing

Antimicrobial susceptibility testing was performed using the Kirby Bauer disc diffusion method.^9^ Susceptibility was tested for the following antimicrobials: ampicillin (10 µg), amoxicillin/clavulanate (20/10 µg), cefoxitin (30 µg), ceftriaxone (30 µg), ceftazidime (30 µg), cefotaxime (30 µg), gentamicin (10 µg), amikacin (30 µg), ciprofloxacin (5 µg), trimethoprim/sulfamethoxazole (1.25/23.75 µg), chloramphenicol (30 µg), imipenem (10 µg), ertapenem (10 µg), meropenem (10 µg), (all Oxoid, UK), eravacycline (20 µg), imipenem/relebactam (10/25 µg) meropenem/vaborbactam (30 µg) and cefiderocol (30 µg) (all MAST, UK).

MIC was determined by the agar dilution method for imipenem and meropenem (Sigma, USA), ETEST strips (bioMérieux, France) for ertapenem, and the broth microdilution method for colistin (Sigma).^10^ Antimicrobial susceptibility testing interpretation is based on CLSI guidelines,^11^ except for colistin, eravacycline, meropenem/vaborbactam and imipenem/relebactam, which were based on current EUCAST breakpoints (https://www.eucast.org/fileadmin/src/media/PDFs/EUCAST_files/Breakpoint_tables/v_12.0_Breakpoint_Tables.pdf).

### WGS

Sequencing was performed in-house for ECO-1 on the Illumina MiSeq short-read sequencing platform with 2 × 250 bp paired-end reads. DNA was extracted using Quick-DNA™ Miniprep Kit (Zymo research, USA), and libraries prepared using the Illumina DNA prep kit (Illumina, USA). The other two strains (ECO-2 and -3) were sequenced commercially at MicrobesNG (https://microbesng.com/) using the Illumina platform. Raw read sequence quality was checked using FASTQC v0.11.9 and trimmed using Trimmomatic v0.39.^12^ Downstream analysis using trimmed reads was performed using Center for Genomic Epidemiology (CGE) tools (http://www.genomicepidemiology.org/). MLST was performed using MLST v2.0^13^ using the Enterobase database.^14^ Resistance genes and point mutations associated with drug resistance in *E. coli* were detected using ResFinder v4.1,^15–17^ while virulence genes and *E. coli* serotypes were detected using VirulenceFinder v2.0^18^ and SerotypeFinder v2.0,^19^ respectively. The WGS data are available in GenBank BioProject PRJNA899445.

### Fitness and virulence assays

#### Growth curve assay

The assay was adapted from previously published methods.^20,21^ Overnight cultures of the strains were prepared in normal saline to a 0.5 McFarland suspension and diluted 1:1000 in either fresh standard LB media (standard condition) or 5% w/v sodium chloride-supplemented LB media (stressed condition). The mixture was incubated at 37°C in triplicate in a microplate reader (Synergy H1, BioTek, USA) and growth was measured every 30 min for 15 h at an OD of 600 nm (OD_600_), with agitation for 5 min prior to each measurement. The assay was performed on three separate days and the averages used to generate growth curves.

#### In vitro mixed growth competition assay

The assay measured the *in vitro* competition between carbapenem-resistant and -susceptible strains from CSF and was adapted from previously published protocols.^20,22,23^ The strains were each grown to exponential phase (4 h) in standard LB or 5% w/v salt-supplemented LB media and incubated at 35°C ± 2°C. The exponentially growing cells were diluted to 0.5 McFarland suspension and 10 µL each of resistant and susceptible strain was added to 10 mL of fresh medium (standard or 5% salt media). The mixture was incubated at 37°C while shaking at 180 rpm for 24 h. Serial 10-fold dilutions of the mixture were plated on Mueller–Hinton agar (MHA) plates without and with 0.25 mg/L meropenem and incubated overnight at 35°C ± 2°C. The cfu/mL for the resistant strain, ECO-2, was calculated from the meropenem-containing plate, and the cfu/mL for the susceptible strain, ECO-1, was determined by subtracting the cfu from plates with antibiotic from those without. The competition index (CI) was defined as the ratio between cfu/mL values of carbapenem-resistant and -susceptible strains. The CI values were calculated for each independent competition assay performed on three separate days and the mean CI determined.

#### Serum bactericidal assay

Normal human serum from healthy volunteers was pooled and frozen at −70°C until use. The assay was adapted from published methods.^21,24,25^ Strains were grown overnight in standard LB broth, then standardized to 0.5 McFarland and grown at 35°C ± 2°C for 4 h to achieve the exponential growth state. One millilitre of the growing cells was aliquoted and spun at 10 000 rpm for 10 min to pellet the bacteria. The pellet was resuspended and washed twice with PBS. The cells were reconstituted in 1 mL of PBS, and 100 µL was added to 900 µL of 40% serum in sterile PBS. The mixture was incubated at 37°C for 3 h with shaking at 180 rpm. Immediately and at 45 min intervals over the 3 h period, an aliquot was serially diluted 10-fold, plated on MHA and incubated overnight at 35°C ± 2°C, and viable cfu/mL value was determined. The assay was performed on three separate days and the mean used to determine serum sensitivity of the strains.

## Results

### Hospital-acquired strains were highly resistant, including to newer antimicrobials

At admission, the first strain identified as a community-acquired *E. coli* strain, ECO-1, was susceptible to all drugs tested except ampicillin and amoxicillin/clavulanate, to which it had reduced susceptibility (intermediate) (Table 1). The reinfection episode was due to hospital-acquired MDR isolates, (ECO-2 from CSF and ECO-3 from blood), which were susceptible to gentamicin, amikacin, chloramphenicol and colistin. The MDR strains were found to be resistant to eravacycline, cefiderocol, imipenem/relebactam and meropenem/vaborbactam, despite no known exposure to any of these compounds.

**Table 1.** Antimicrobial susceptibility profile, resistance genes and point mutations in E. coli strains from recurrent meningitis in adult patient

Drug class	Drug tested	ECO-1		ECO-2		ECO-3	
		AST	Resistance gene(s)	AST	Resistance gene(s)/point mutations	AST	Resistance gene(s)/point mutations
Aminoglycosides	Gentamicin	S	None identified	S	*aadA2, aac(6′)-Ib-cr, aac(3)-lla*	S	*aadA2, aac(6′)-Ib-cr*
	Amikacin	S		S		S	
Carbapenems	Meropenem	S	None identified	R	*bla*_NDM-5_, *bla*_CTX-M-15_, *bla*_OXA-1_	R	*bla*_NDM-5_, *bla*_CTX-M-15_, *bla*_OXA-1_
	Imipenem	S		R		R	
	Ertapenem	S		R		R	
	Imipenem/relebactam	S		R		R	
	Meropenem/vaborbactam	S		R		R	
Cephalosporins	Ceftriaxone	S		R		R	
	Ceftazidime	S		R		R	
	Cefotaxime	S		R		R	
	Cefiderocol	S		R		R	
Cephamycins	Cefoxitin	S		R		R	
Penicillins	Ampicillin	I		R		R	
	Amoxicillin/clavulanate	I		R		R	
Quinolones	Ciprofloxacin	S	None identified	R	*gyrA* (S83L, D87N), *parC* (S80I), *parE* (S458A)	R	*gyrA* (S83L, D87N), *parC* (S80I), *parE* (S458A)
Phenicols	Chloramphenicol	S	None identified	S	*catB3*	S	*catB3*
Folate pathway antagonists	Sulfamethoxazole/trimethoprim	S	*sul2*	I	*dfrA12, sul1*	R	*dfrA12, sul1*
Polymyxins	Colistin	S	None identified	S	None identified	S	None identified
Tetracyclines	Tetracycline	ND	*tet*(B)	ND	*tet*(A)	ND	*tet*(A)
	Eravacycline	S		R		R	
Macrolides	Azithromycin	ND	None identified	ND	*mph*(A)	ND	*mph*(A)
Others	—	—	sitABCD	—	*mdf*(A), *qacE*	—	*mdf*(A), *qacE*

Antimicrobial susceptibility testing interpretation is based on CSLI guidelines for all drugs except colistin, eravacycline, meropenem/vaborbactam and imipenem/relebactam, which were based on current EUCAST breakpoints. S, susceptible; R, resistant; I, intermediate. ND, not done.

The hospital-acquired strains possessed several resistance genes including β-lactamases *bla*_NDM-5_, *bla*_CTX-M-15_ and *bla*_OXA-1_, aminoglycoside resistance genes *aadA2*, *aac(6′)-Ib-cr* and *aac(3)-lla* and genes conferring resistance to other antimicrobials and cleaning compounds *catB3*, *dfrA12*, *sul1*, *tet*(A), *mph*(A), *mdf*(A) and *qacE* (Table 1). In addition, point mutations in gyrase (*gyrA*) and topoisomerase genes (*parC* and *parE*) conferring resistance to fluoroquinolones were present. The ECO-1 strain had two resistance-associated genes, *sul2* and *tet*(B), which confer resistance to sulfamethoxazole and tetracycline, respectively. The strain was susceptible to sulfamethoxazole/trimethoprim *in vitro* despite the presence of *sul2*. Phenotypic susceptibility to tetracycline was not tested.

### Community-acquired strain was unrelated to hospital-acquired strains

Genomic analysis revealed the community-acquired strain (ECO-1) to be ST88, serotype O8:H17, while the hospital-acquired CSF (ECO-2) and bacteraemia strains (ECO-3) were both ST167, serotype O101:H5. There were also differences in the plasmids found in strains with one plasmid, Col8282, detected in the community-acquired strain and four plasmids, Col(BS512), ColpVC, IncFIA and IncFII, detected in the hospital-acquired strains.

### Hospital-acquired strain was less virulent and less fit than community-acquired strain

The community-acquired strain had additional virulence genes compared with the hospital-acquired strain, with the presence of genes involved in iron acquisition (*iucC*, *iutA*, *fyuA*, *ireA*, *iroN*, *sitA*, *irp2*), serum resistance (*iss*, *traT*), toxicity (*hlyF*), adhesion (*papC*, *papA*_F20, *lpfA*), outer membrane protease (*ompT*), bacteriocin (*cvaC*) and miscellaneous genes (*hra*, *tsh*, *etsC* and *mchF*). The resistant strains possessed far fewer virulence genes, with only *iss* and *traT* (involved in serum resistance) and *hra*, a heat-resistant agglutinin, identified in the genome. These three genes were also present in the community-acquired strain. None of the strains possessed the Shiga toxin gene.

**Figure 1.**
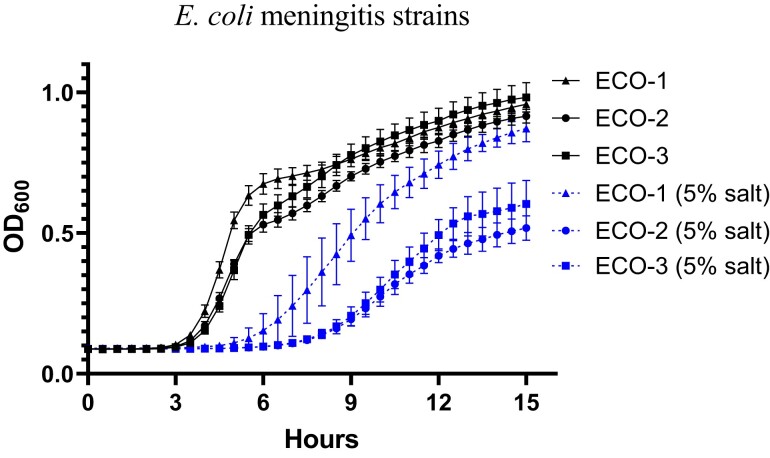
Growth curves of *E.coli* meningitis strains in standard and salt supplemented (5% w/v) LB media. Carbapenem-susceptible *E. coli* strain from CSF, ECO-1 (filled triangle), and carbapenem-resistant strains from CSF, ECO-2 (filled circle) and blood, ECO-3 (filled square) were grown at 37°C and OD_600_ measured at 30 min intervals. The strains were grown in triplicate on three separate days with the mean plotted and error bars indicating SD.

**Figure 2.**
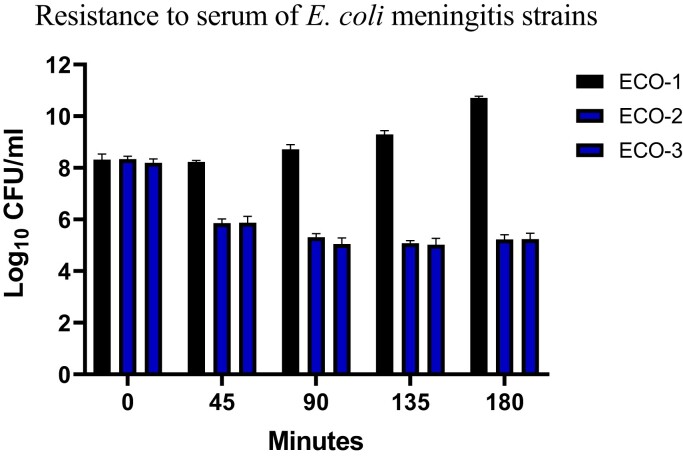
Survival of carbapenem-susceptible and resistant *E. coli* strains in healthy human serum. Strains were incubated in 40% pooled normal human serum at 37°C for 3 h. Aliquots were taken every 45 min and colonies enumerated by plating. Three separate experiments were performed for each isolate and the mean and SD (error bars) plotted for each strain.

In individual growth curves in standard LB broth, the susceptible strain, ECO-1, grew slightly faster than ECO-2 and ECO-3 (resistant strains), and the difference was amplified in 5% salt media, with detectable bacterial amplification at 5.5 h for ECO-1 versus 8.5 h for ECO-2 and ECO-3 (Figure 1). Pairwise competition between the CSF strains demonstrated that under standard conditions, ECO-2 was less fit, with a mean CI of 0.695 (SD = 0.25); however, in 5% salt it was the ECO-1 strain that was less fit (mean CI 2.33, SD = 1.44). ECO-2 and ECO-3 showed reduced survival in normal human serum compared with ECO-1, with a reduction in viable bacterial count from 8 log_10_ cfu/mL to less than 6 log_10_ cfu/mL by 45 min and through the assay, compared with ECO-1, which was unaffected at 45 min and increased to 10 log_10_ cfu/mL by 180 min (Figure 2).

## Discussion

Here we present an interesting case of recurrent meningitis in an adult with two distinct *E. coli* strains, one of which was carbapenem resistant. To the best of our knowledge, this is the first non-surgical case where reinfection has been reported with an entirely different strain of *E. coli*. Spontaneous community-acquired *E. coli* meningitis in immunocompetent adults is rare, attributed to predisposing risk factors and/or occurring secondary to a distant infection. Though published data on adult meningitis in Africa are limited, non-meningococcus and non-pneumococcus bacteria including *E. coli* accounted for 10% of all cases in adolescents and adults reported from 1990 to 2013 in Malawi.^26^

The community- and hospital-acquired strains had different antibiograms, suggesting that they were unrelated. This was confirmed by WGS, with the initial community-acquired strain being ST88 and the hospital-acquired isolate belonging to an unrelated ST, ST167. They also had different resistance and virulence gene profiles and possessed different plasmid types. This was, therefore, a new infection rather than *in vivo* acquisition of resistance in the initial strain.

Previous studies have identified chronic alcoholism as a risk factor for Gram-negative meningitis.^4^ This may explain the initial presentation in this patient, who had a history of excess alcohol intake, although he had not progressed to liver impairment. While no other risk factors were identified, he had complained of symptoms suggestive of gastroenteritis prior to his admission. It is therefore possible that there was an intrabdominal source that was not identified during his stay, as no gastrointestinal investigations were carried out because his symptoms were ascribed to meningitis and sepsis.

Alternatively, the patient may have had a mixed infection consisting of both carbapenem-resistant and -susceptible strains, acquired prior to admission to hospital. If the resistant strain was present in low density it may have been missed during selection of a single colony for susceptibility testing. Treatment with a broad-spectrum cephalosporin may have resulted in initial control of the infection, reduction in bacterial load and subsequent improvement in the patient’s condition, while concurrently providing the selective pressure that allowed the resistant strain to survive. However, given the patient’s lack of prior hospital exposure, this is less likely given the relative rarity of carbapenem resistance in the community in Kenya, and the MDR strain was therefore more likely to be hospital acquired.

The community-acquired strain possessed multiple virulence genes, which may explain the initial infection. A recent study in France showed that patients who died of *E. coli* meningitis were more likely to have been infected with a more virulent strain compared with survivors.^8^ Moussiegt *et al.*^8^ also reported that acquired resistance genes in the meningitis strains was rare, a phenomenon observed in the initial infecting strain in our case study. The hospital-acquired strain possessed very few virulence genes, and the lack of virulence was confirmed *in vitro,* with reduced susceptibility to serum from healthy humans. In competition assays, it was less fit than the community-acquired strain, except in pairwise competition in 5% salt, where there was no significant difference in growth between the strains, suggesting similar responses to this specific stressor.

Despite this, it was able to infect the patient with a lethal outcome, suggesting that in this case, host factors played a significant role in the pathogenesis of infection in this patient. We postulate, therefore, that debilitation due to chronic alcohol intake and poor nutrition, together with a possible intra-abdominal source of infection, caused initial infection in this patient, who unfortunately become colonized with a carbapenem-resistant strain during his hospital stay. Those same risk factors, together with the selective pressure from the cephalosporin administered for his first infection, subsequently predisposed him to a second infection, this time with the resistant strain. Additionally, although the patient did not have HIV, underlying immune dysfunction due to poor nutrition^27,28^ and chronic alcohol use^29^ cannot be ruled out, with the carbapenem-resistant organism behaving as an opportunistic pathogen.

Discordance between genotypic and phenotypic results was observed for the aminoglycosides, chloramphenicol and sulfamethoxazole/trimethoprim. The *catB3* gene in the resistant strain, which confers chloramphenicol resistance, had only 70% coverage to the reference gene used in ResFinder and thus may not have yielded a fully functioning protein; however, in the case of aminoglycosides the genes detected may confer resistance to other aminoglycosides such as spectinomycin while the presence of *aac(6′)-Ib-cr* does not necessarily confer resistance to amikacin, gentamicin or ciprofloxacin.^30^ The reasons for the susceptibility to sulfamethoxazole/trimethoprim despite the presence of the *sul2* gene in the susceptible *E. coli* strain remain unknown, though likely causes may include changes in the promoter region affecting expression or the less-known antibiotic resistance gene-silencing concept previously reported in animal studies.^31^ In some strains, drug susceptibility was observed despite the plasmid possessing intact ORFs and promoter regions for *bla*_OXA-2_, *aadA1*, *sul1* and *tet*(A) genes similar to WTs. In such cases, the transcripts were found to be lacking, suggesting that resistance gene expression could be switched off, though the mechanisms are unknown. However, this phenomenon was potentially reversible when the plasmid was introduced into a new host bacterium, implying that chromosomal factors rather than plasmid ones were responsible.

The patient was treated with chloramphenicol but died 2 days later. While a partial chloramphenicol resistance gene was identified in the resistant strain, phenotypic susceptibility testing predicted *in vivo* efficacy, but by the time testing was complete he had been on ineffective therapy with ceftriaxone for 3 days. An alternative treatment regimen with a combination of chloramphenicol and amikacin may have been beneficial in this case; however, even with treatment that is concordant with *in vitro* susceptibility, more than a third of spontaneous *E. coli* meningitis patients die.^4,5^ It is likely that the underlying factors contributing to initial infection also contributed to death in this case.

A final, important aspect of this case is the extensive resistance observed in the hospital-acquired strain. Carbapenems are generally unavailable in the public sector in Kenya,^32,33^ although they are widely used in private hospitals,^34^ and there was allegedly no history of prior antimicrobial use in this patient. Carbapenems have only been available in the hospital formulary in Kilifi since 2020, although surveillance data indicate the presence of carbapenem-resistant organisms since 2005 (A. V. Amulele, A. M. Arara, E. W. Machanja, D. W. Wareham, J. A. Berkley and N. C. Gordon, unpublished data).

Even more concerning is the resistance to eravacycline, cefiderocol and the newer β-lactam/inhibitor combinations. To the best of our knowledge, these drugs are not available in Kenya; however, resistance to eravacycline and tigecycline (closely related to eravacycline) due to the presence of plasmid-encoded variants of the *tet*(X) gene: *tet*(X4)^35,36^ and *tet*(X5),^37^ as well as mutations in the efflux pump AdeABC,^38^ have been reported in Gram-negative bacteria from Asia. Resistance to cefiderocol has been associated with mutations in siderophore receptor gene, *cirA*, in Enterobacterales,^39, 40^ with a recent study^41^ reporting the concomitant presence of β-lactamases such as *bla*_NDM_ and mutations in *cirA* resulting in resistance to the antibiotic. We did not detect the presence of *tet*(X) genes in the MDR strains but it is likely that mutations in the efflux pumps or the siderophore may be involved in resistance.

The presence of resistance to drugs not currently in use in the country is deeply concerning and illustrates the ongoing challenges of antimicrobial resistance. Surveillance to detect resistance to new antibiotics is necessary and a One Health approach that incorporates the animal and agricultural sectors can help detect reservoirs for MDR bacteria with clinical implications.
